# Supplementary material for: Recombinant Salmonella gallinarum (S. gallinarum) Vaccine Candidate Expressing Avian Pathogenic Escherichia coli Type I Fimbriae Provides Protections against APEC O78 and O161 Serogroups and S. gallinarum Infection
Source: Vaccines (Basel). 2023 Nov 28;11(12):1778. doi: 10.3390/vaccines11121778 (PMC10747928; doi:10.3390/vaccines11121778)
Supplement: Supplementary file 1 [file vaccines-11-01778-s001.zip › Figure S1. Average anti-Salmonella peg antibody agglutination titers of chickens at different weeks post inoculation.pdf]

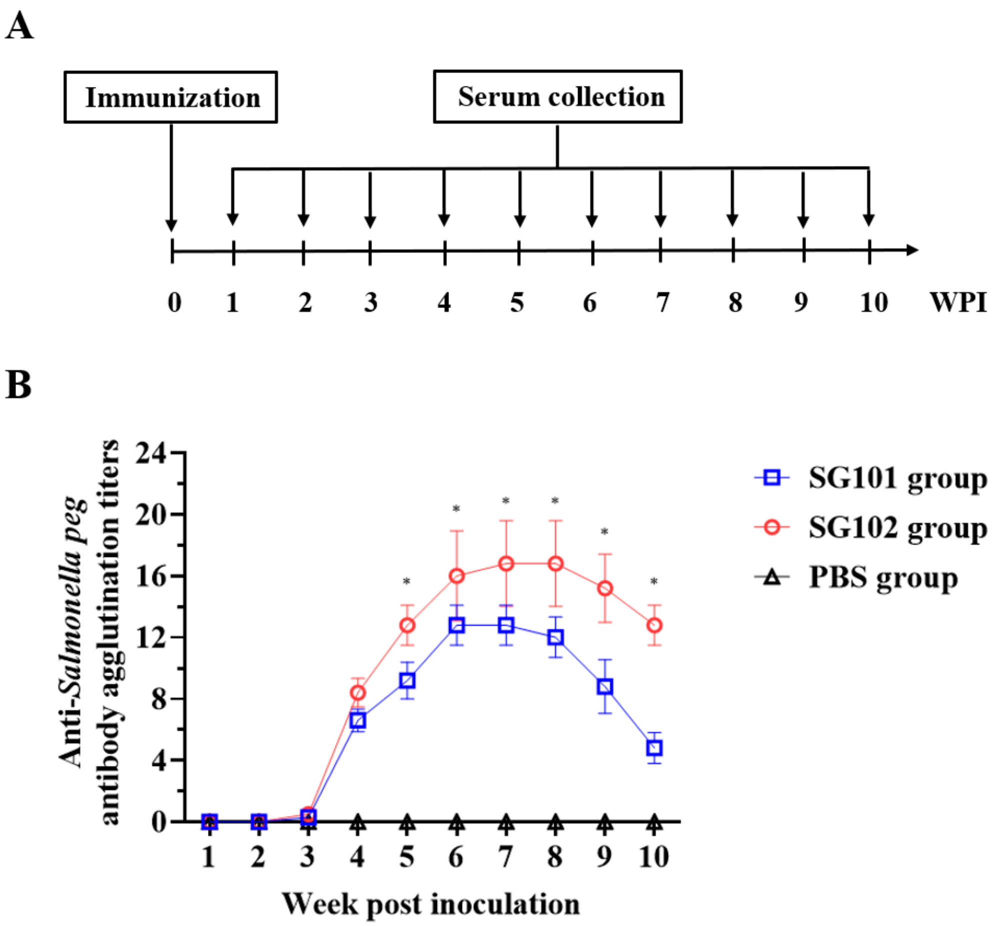

Figure S1. Average anti-*Salmonella peg* antibody agglutination titers of chickens at different weeks post inoculation. (A) SG101 and SG102 group were orally inoculated with 5×10^9 CFU of SG101 or SG102 strain in 200 μL of PBS, respectively, while the PBS group was only orally inoculated with 200 μL of PBS. The serum samples of all chickens were collected at 1, 2, 3, 4, 5, 6, 7, 8, 9 and 10 wpi. (B) Anti-*Salmonella peg* antibody agglutination titers of chickens were detected by agglutination reactions. Negative agglutination reactions were identified in serum collected from the PBS group at each week post inoculation. Positive agglutination reactions started to be identified at 3 wpi and still could be detected at 10 wpi from SG101-immunized and SG102-immunized chickens. Average anti-*Salmonella peg* antibody agglutination titer of SG102 group were significantly higher than that of SG101 group at 5, 6, 7, 8, 9 and 10 wpi. The data were recorded as the mean ± SEM. Average antibody agglutination titers of SG101 and SG102 group were compared by using the unpaired Student's *t* test. * $P < 0.05$.
