# Supplementary material for: Recombinant Salmonella gallinarum (S. gallinarum) Vaccine Candidate Expressing Avian Pathogenic Escherichia coli Type I Fimbriae Provides Protections against APEC O78 and O161 Serogroups and S. gallinarum Infection
Source: Vaccines (Basel). 2023 Nov 28;11(12):1778. doi: 10.3390/vaccines11121778 (PMC10747928; doi:10.3390/vaccines11121778)
Supplement: Supplementary file 1 [file vaccines-11-01778-s001.zip › Figure S2. Protection effects against S. gallinarum challenge.pdf]

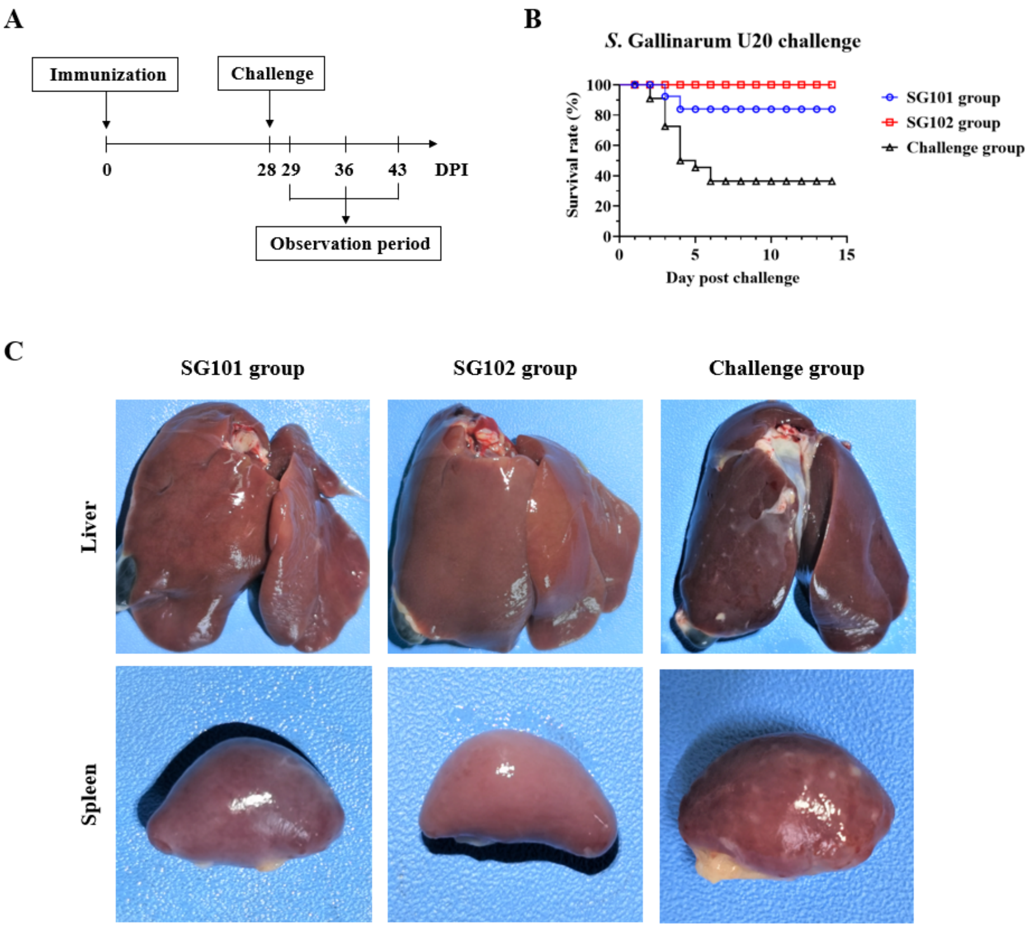

Figure S2. Protection effects against *S. gallinarum* challenge. (A) Chickens were immunized by SG101, SG102 and PBS suspensions, respectively. At 28 days after the inoculation, all chickens were challenge with the virulent *S. gallinarum* strain U20. Chicken deaths were recorded daily during the 14 days of observation period. (B) The survival rate for the SG101, SG102 and challenge group after challenge with the U20 strain. (C) Pathological changes of the liver and spleen in chickens from three groups after challenge with the U20 strain. The differences in survival rates between SG101 and SG102 groups were determined by the log-rank sum test.
