# Supplementary material for: Recombinant Salmonella gallinarum (S. gallinarum) Vaccine Candidate Expressing Avian Pathogenic Escherichia coli Type I Fimbriae Provides Protections against APEC O78 and O161 Serogroups and S. gallinarum Infection
Source: Vaccines (Basel). 2023 Nov 28;11(12):1778. doi: 10.3390/vaccines11121778 (PMC10747928; doi:10.3390/vaccines11121778)
Supplement: Supplementary file 1 [file vaccines-11-01778-s001.zip › Table S1. Primers used in this study.pdf]

Table S1. Primers used in this study.

Primers	Sequence (5' →3')
P1-C1	cacctcggcgacacctccaccggcacgctacaggacgcttttgatctggTGTGTAGGCTGGAGCTGCTTCGT
P2-C2	gccgccggggttaattcgcgcatagtgatatccgatcgttcggcacCCATATGAATATCCTCCTTAG
P3	cggctctgttctcatgcaac
P4	gccaactggcgcagcattcg
pBR322-*fim* NheI F	cGCTAGCatgaaaattaaaactctgg
pYA3342-*fim* NcoI F	gcgcCCATGGatgaaaattaaaactctgg
pBR322/pYA3342-*fim* SalI R	cgcGTCGACttattgataaacaaaagtcac
