# Supplementary material for: Recombinant Salmonella gallinarum (S. gallinarum) Vaccine Candidate Expressing Avian Pathogenic Escherichia coli Type I Fimbriae Provides Protections against APEC O78 and O161 Serogroups and S. gallinarum Infection
Source: Vaccines (Basel). 2023 Nov 28;11(12):1778. doi: 10.3390/vaccines11121778 (PMC10747928; doi:10.3390/vaccines11121778)
Supplement: Supplementary file 1 [file vaccines-11-01778-s001.zip › Table S2. Anti-Salmonella peg antibody agglutination titers of chickens with different immunity periods.pdf]

Table S2. Anti-*Salmonella peg* antibody agglutination titers of chickens with different immunity periods.

Serial Number [a]	Agglutination antibody titers									
	1 wpi [b]	2 wpi	3 wpi	4 wpi	5 wpi	6 wpi	7 wpi	8 wpi	9 wpi	10 wpi
SG101 immunized group										
1	N [c]	N	1:1	1:8	1:8	1:8	1:16	1:8	1:4	N
2	N	N	N	1:4	1:16	1:16	1:8	1:8	1:8	1:4
3	N	N	1:1	1:8	1:8	1:16	1:16	1:16	1:16	1:8
4	N	N	N	1:8	1:8	1:16	1:16	1:16	1:16	1:8
5	N	N	N	1:8	1:8	1:16	1:16	1:8	1:8	1:4
6	N	N	N	1:8	1:8	1:16	1:16	1:16	1:16	1:8
7	N	N	N	1:4	1:16	1:16	1:16	1:16	1:16	1:8
8	N	N	1:1	1:8	1:8	1:8	1:16	1:16	1:8	1:4
9	N	N	N	1:4	1:8	1:8	1:8	1:8	1:4	1:4
10	N	N	N	1:2	1:8	1:8	1:8	1:8	1:4	N
SG102 immunized group										
11	N	N	1:1	1:8	1:8	1:8	1:16	1:16	1:16	1:16
12	N	N	N	1:8	1:8	1:8	1:8	1:8	1:8	1:8
13	N	N	1:1	1:16	1:16	1:16	1:16	1:32	1:16	1:16
14	N	N	N	1:4	1:8	1:8	1:8	1:8	1:8	1:4
15	N	N	N	1:8	1:16	1:32	1:32	1:32	1:32	1:16
16	N	N	N	1:8	1:8	1:16	1:16	1:16	1:16	1:16
17	N	N	1:1	1:8	1:16	1:16	1:16	1:16	1:16	1:16
18	N	N	1:1	1:8	1: 16	1:32	1:16	1:16	1:16	1:8
19	N	N	N	1:8	1:8	1:8	1:8	1:8	1:8	1:8
20	N	N	1:1	1:8	1:16	1:16	1:32	1:16	1:16	1:16
PBS group										
21	N	N	N	N	N	N	N	N	N	N
22	N	N	N	N	N	N	N	N	N	N
23	N	N	N	N	N	N	N	N	N	N
24	N	N	N	N	N	N	N	N	N	N
25	N	N	N	N	N	N	N	N	N	N
26	N	N	N	N	N	N	N	N	N	N
27	N	N	N	N	N	N	N	N	N	N
28	N	N	N	N	N	N	N	N	N	N
29	N	N	N	N	N	N	N	N	N	N
30	N	N	N	N	N	N	N	N	N	N

[a] Serial number of the chicken.

[b] Week post inoculation.

[c] Negative reaction.
